# The effects of time-restricted eating for patients with nonalcoholic fatty liver disease: a systematic review

**DOI:** 10.3389/fnut.2023.1307736

**Published:** 2024-01-04

**Authors:** Xiaoxiao Lin, Shuai Wang, Jinyu Huang

**Affiliations:** Affiliated Hangzhou First People's Hospital, Zhejiang University School of Medicine, Zhejiang University, Hangzhou, Zhejiang, China

**Keywords:** nonalcoholic fatty liver disease, time-restricted eating, systematic review, efficacy, live parameters

## Abstract

Nonalcoholic fatty liver disease (NAFLD) represents a significant global health concern. Numerous investigations have explored the implications of time-restricted eating (TRE) in the management of NAFLD. Therefore, the objective of our study was to conduct a systematic review to summarize and analyze all randomized controlled trials (RCTs) of TRE for patients with NAFLD. A thorough literature search was executed across Embase, Cochrane Library, and PubMed databases, covering all records from their inception until 1 September 2023. All clinical studies of TRE for NAFLD were summarized and analyzed. Our systematic review included four RCTs, encompassing a total of 443 NAFLD patients. These studies varied in sample size from 32 to 271 participants. The TRE intervention was consistently applied in an 8-h window, over durations ranging from 4 weeks to 12 months. The findings suggest that TRE could offer several health benefits for NAFLD patients, such as improved liver health indicators like liver stiffness and intrahepatic triglyceride (IHTG) levels. Consequently, TRE appears to be a promising dietary intervention for NAFLD patients. However, it is premature to recommend TRE for patients with NAFLD. The existing body of research on the effects of TRE in NAFLD contexts is limited, underscoring the need for further high-quality studies to expand our understanding of TRE’s benefits in treating NAFLD. Ongoing clinical trials may provide more insights into the effects of TRE in NAFLD.

## 1 Introduction

Nonalcoholic fatty liver disease (NAFLD) is a prevalent global health concern, affecting approximately 20 to 30% of individuals worldwide, thus representing a significant public health challenge (1–3). Defined by the accumulation of fat in over 5% of hepatocytes, and unrelated to viral hepatitis, excessive alcohol consumption, or other causes such as steatogenic medications, NAFLD is a multifaceted condition influenced by a combination of genetic predispositions and environmental factors (2, 3). Recent research indicates a global incidence rate of NAFLD of 4,613 cases per 100,000 person-years, with higher rates observed in individuals who are obese or overweight and in men (3). The disease poses a substantial risk for liver-related morbidity and mortality, along with metabolic comorbidities, underscoring the need for increased attention from health policymakers, primary care physicians, and specialists (2). Notably, NAFLD is comorbid in over 70% of individuals with obesity or diabetes and is commonly associated with type 2 diabetes mellitus (T2DM), hyperlipidemia, obesity, and certain cardiovascular diseases (4–7). Recent research has elucidated the potential of specific dietary interventions and supplements in ameliorating liver and metabolic parameters in patients with NAFLD (8–13).

In recent years, intermittent fasting (IF) diets have gained significant popularity due to their clinical benefits, including improvements in metabolic factors and the facilitation of weight loss (14–16). These diets are primarily categorized into three types: time-restricted eating (TRE), which involves consuming meals within a consistent daily window (typically 8 h); alternate-day fasting (ADF), characterized by alternating fasting and feasting days; and the 5:2 diet, which involves 5 days of normal eating and two fasting days per week (17–22). These dietary approaches have demonstrated benefits in managing hyperglycemia (23) and nonalcoholic fatty liver disease (NAFLD) (24). TRE may have some benefits for healthy individuals, especially early TRE (25–27). Additionally, specific forms of IF, such as Ramadan fasting and the fasting-mimicking diet, have been identified as potentially beneficial in managing obesity (28, 29), NAFLD (30, 31), and even in cancer therapy (32, 33). Among these, TRE has been increasingly emphasized for its potential to enhance adherence, improve various cardiometabolic parameters, and contribute to overall health throughout the lifespan (34–40). TRE is a dietary strategy within the broader intermittent fasting paradigm, focusing on limiting food intake to a specific time window, typically 8 h each day. This approach contrasts with traditional dietary methods that concentrate on caloric or food type restrictions. The primary premise of TRE is that aligning eating patterns with circadian rhythms can optimize physiological processes, potentially leading to improved metabolic health, weight management, and reduced risk of chronic diseases. A systematic scoping review explored the feasibility and safety of TRE for individuals with T2DM, prediabetes, obesity, and who were overweight, and found that TRE was acceptable, implementable, and safe (41). Previous studies have demonstrated that TRE may be beneficial for some disorders, including diabetes (42, 43), prediabetes (44, 45), polycystic ovary syndrome (PCOS) (46, 47), metabolic syndrome (48, 49), and multiple sclerosis (50). In addition, several studies have explored the effects of TRE in NAFLD. Therefore, the objective of our study was to conduct a systematic review to summarize and analyze all clinical studies on the effects of TRE for patients with NAFLD on body composition, cardiometabolic and inflammatory biomarkers, and NAFLD parameters.

## 2 Methods

### 2.1 Search strategy

The methodology for this systematic review was meticulously predefined and registered on the INPLASY platform (Registration ID: 202380036). This review was executed in accordance with the methodologies recommended by the Cochrane Collaboration and adhered to the PRISMA guidelines for reporting outcomes (51). A thorough literature search was conducted across the Embase, PubMed, and Cochrane databases, spanning from their inception to 1 September 2023. The search incorporated the following terms: (“Nonalcoholic Fatty Liver” OR “Nonalcoholic Fatty Liver Disease” OR “Fatty Liver, Nonalcoholic” OR “Liver, Nonalcoholic Fatty” OR NAFLD OR “Non-alcoholic Fatty Liver Disease” OR “Nonalcoholic Fatty Livers” OR “Nonalcoholic Steatohepatitis” OR “Steatohepatitis, Nonalcoholic” OR “metabolic dysfunction-associated fatty liver disease” OR MAFLD) AND (“time restricted” or “time restricted feeding” or “time restricted eating” or time-restricted or “time-restricted feeding” or “time-restricted eating” OR “Eating, Time Restricted” OR “Time Restricted Feedings” OR TRE OR intermittent OR “Intermittent Fasting” OR “Fasting, Intermittent” OR “Fasting, Time Restricted” OR “Feeding, Time Restricted” OR “Time Restricted Eating” OR “Time Restricted Fasting” OR “Time Restricted Feeding”). The references in relevant reviews were also searched. In cases where there was uncertainty, the initial literature search and screening were conducted by two authors (LXX and WS), and any ambiguities were resolved through consultation with a third author (HJY).

### 2.2 Inclusion criteria

In accordance with the PICOS framework (52), the inclusion criteria for this systematic review were meticulously established as follows: (P) patients, encompassing individuals diagnosed with NAFLD; (I) intervention, specifically focusing on time-restricted eating (TRE); (C) control, which included several comparative conditions such as calorie restriction (CR), habitual meal timing, alternate-day fasting (ADF), or a standard diet; (O) outcomes, with primary endpoints being the effects of TRE on liver parameters in NAFLD patients and secondary outcomes including alterations in body weight, plasma lipid levels, body composition, insulin resistance, glucoregulatory factors, inflammation parameters (cytokeratin and high-sensitivity C-reactive protein), and the safety profile of TRE; and (S) study type, which was RCTs. Exclusions were made for editorials, duplicates, comments, conference abstracts, and case reports.

### 2.3 Quality assessment and data extraction

For the assessment of randomized controlled trials (RCTs) (53), we employed the Cochrane Collaboration’s Risk of Bias 2 tool to evaluate the quality. Data extraction was carried out independently by two authors (LXX and WS) and was organized into two predefined templates: (1) study characteristics, encompassing details of the first author, publication year, study design, sample size, intervention and control groups, TRE window, main outcomes measured, and principal findings and (2) patient characteristics, detailing the first author, publication year, mean age, body mass index (BMI) in kg/m^2^, waist circumference, percentage of female participants, weight, fat mass, and lean body mass. Additionally, an exploration for ongoing TRE studies involving patients with NAFLD was conducted on the ClinicalTrials.gov website.^1^ The extracted data from these ongoing studies were organized into a predefined format, including study title, NCT number, conditions, interventions, study type, study commencement, locations, and expected completion dates.

## 3 Results

### 3.1 Literature search

**Figure 1:**
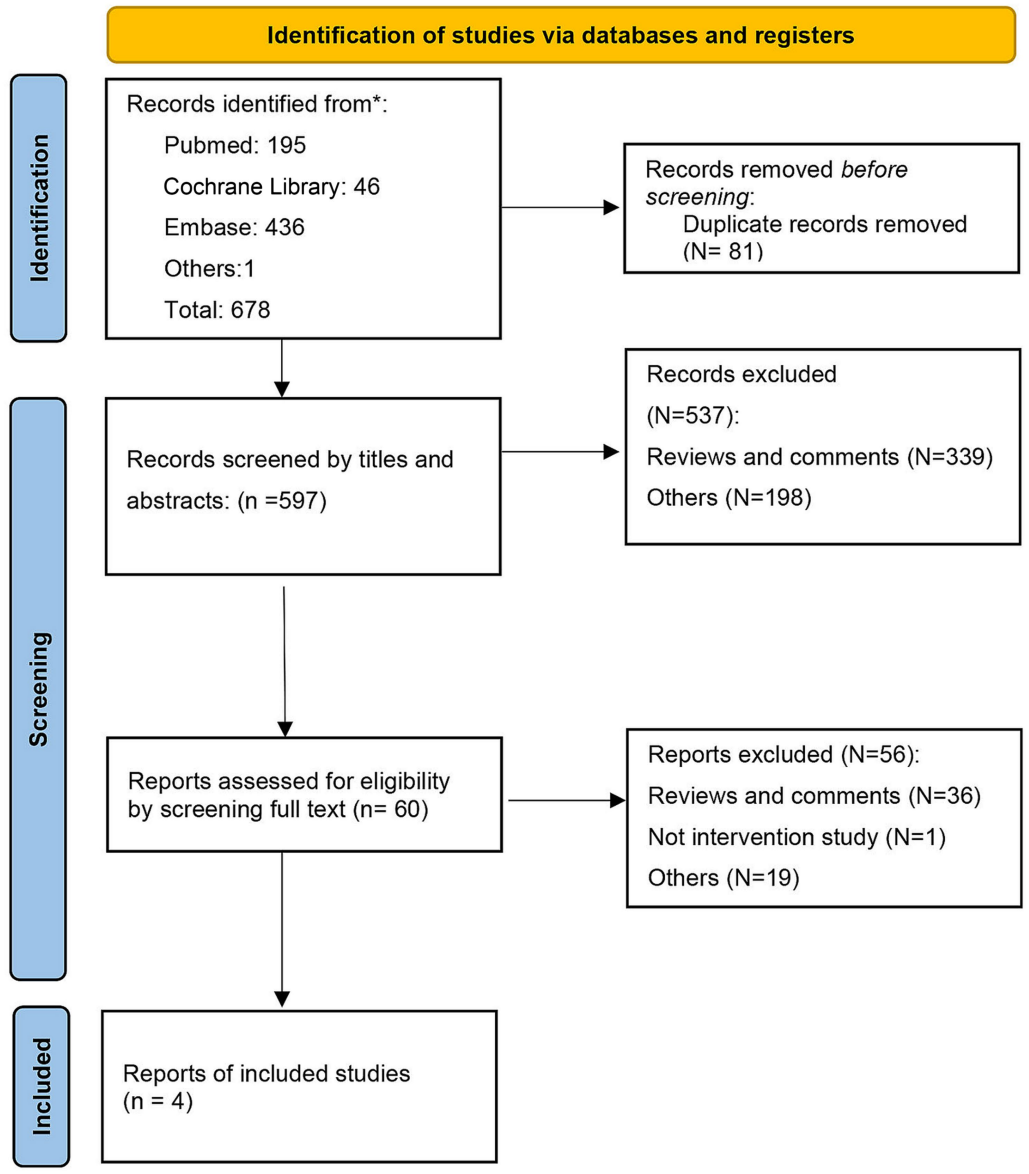
Search flow diagram.

A total of 678 records were found in our primary database search. After excluding duplications, 596 records were screened by title, abstracts, and full text. Finally, 4 RCTs were included in the systematic review (54–57). The search flow diagram is displayed in Figure 1.

### 3.2 Study characteristics and quality assessment

**Figure 2:**
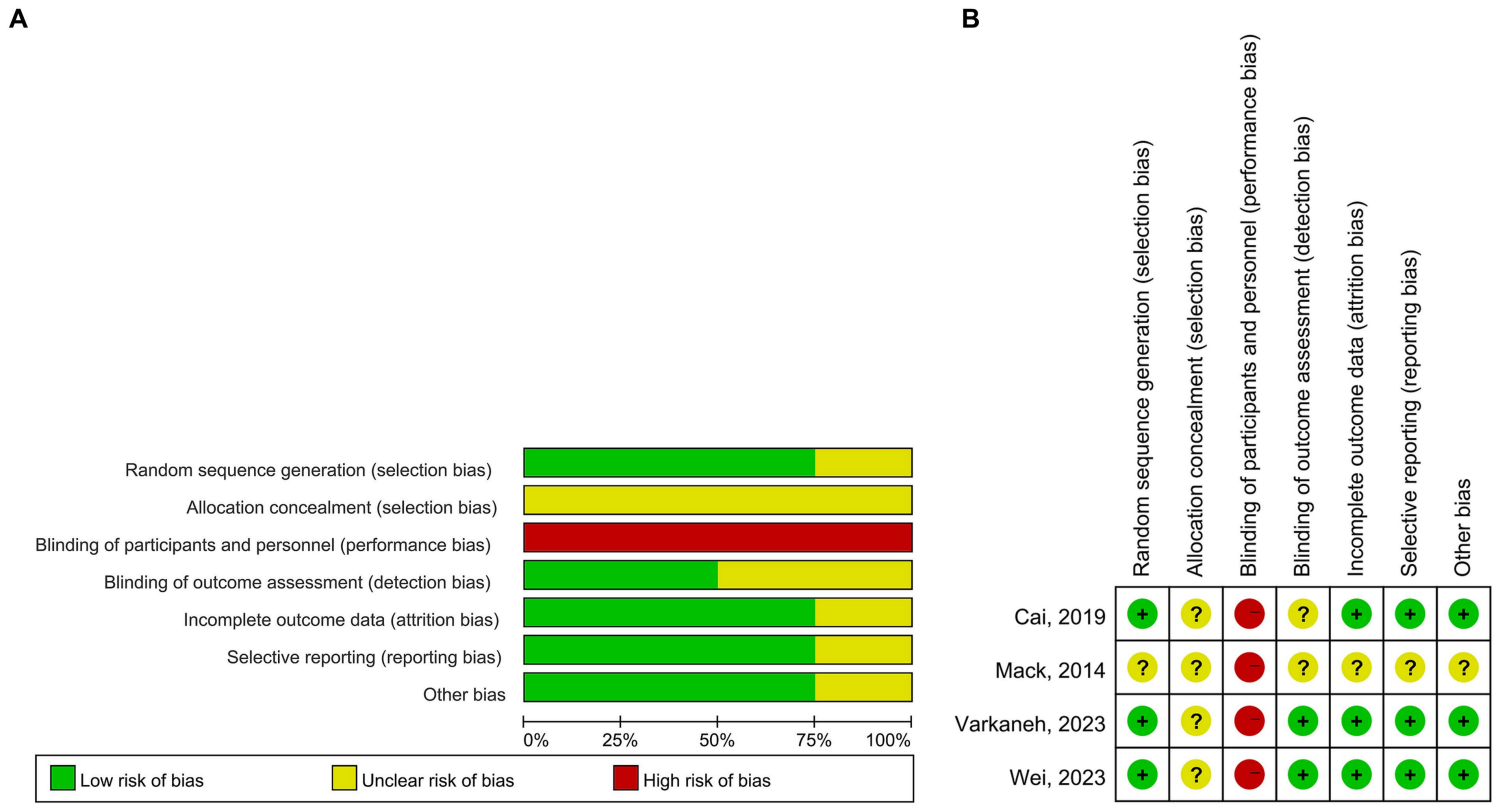
Quality assessment. **(A)** Risk of bias graph and **(B)** risk of bias summary.

A total of 443 patients with NAFLD were included. The sample size ranged from 32 to 271. The TRE windows in all studies were 8 h. The interventions were TRE without CR in two studies, TRE with CR in one study, and TRE with a low-sugar diet in one study. The time of interventions ranged from 4 weeks to 12 months. One study included patients with obesity only (56) and two studies did not (55, 57). The mean age ranged from 32.3 to 41.36 years, the ratios of females ranged from 45.5 to 69.5%, and the mean BMI ranged from 26.76 to 32.2 in TRE groups. Study and patient characteristics are summarized in Tables 1, 2. For quality assessment, three studies reported sequence generation methods, the computer-generated random number sequence was used in two studies, and a block size of six was used in one study. The results of the quality assessment for RCTs are shown in Figure 2.

**Table 1:** Characteristics of the included studies.

Study, year	Sample size	Intervention group	Control group	TRE window	Main measured outcomes	Main findings
Cai et al. (2019) (57)	271	TRE	ADF and a control diet	4 weeks, 8 h consistent eating time daily	Body weight, body composition, and cardiometabolic risk factors, live parameters	TRE can reduce body weight, fat mass, total cholesterol, and serum triglycerides. TRE and ADF were effective diet interventions for patients with NAFLD, achieving improvements in hyperlipidemia and reducing body weight.
Kord-Varkaneh et al. (2023) (55)	52	TRE and a low-sugar diet	Control diet	12 weeks, 8 h consistent eating time daily	Body weight, body composition, cardiometabolic risk factors, glucose homeostasis, liver parameters, and inflammation	TRF plus a low-sugar diet can improve lipid, liver, and inflammatory markers, and reduce adiposity. Therefore, TRF combined with a low-sugar diet could be regarded as a promising non-pharmacologic intervention for patients with NAFLD, and long-term adherence in diverse populations should be considered in further studies.
Wei et al. (2023) (56)	88	TRE plus CR	DCR (habitual meal timing) and CR	12 months, 8 h consistent eating time daily	Body weight, body composition, cardiometabolic risk factors, glucose homeostasis, and liver parameters	TRE and CR reduced the intrahepatic triglyceride (IHTG) content by 8.3 and 8.1%, respectively, at the 6-month assessment. TRE and DCR can significantly reduce body weight, metabolic risk factors, and liver stiffness; however, there was no significant difference between the two groups regarding these factors. In addition, in terms of the safety of TRE, the authors found that there were no significant differences between the TRE plus CR group and the CR group for adverse events, including discomfort in the stomach, appetite change, dyspepsia, constipation, hunger, fatigue, decreased appetite, and dizziness. Their findings supported that caloric restriction should be added to TRE.
Mack et al. (2014) (54)	32	TRE	SC	12 weeks, 8 h TRE	Fat mass, anthropometric and biochemical measurements	TRE is a well-tolerated strategy to treat NAFLD and central adiposity, with significantly greater improvements in transient elastography (liver stiffness and CAP), waist circumference, visceral fat, and insulin resistance compared to standard diet and exercise in this pilot study.

**Table 2:** Characteristics of the patients.

Type	Kord-Varkaneh et al. (2023) (55)		Wei et al. (2023) (56)		Cai et al. (2019) (57)			Mack et al. (2014) (54)	
	TRE (*n* = 22)	Control (*n* = 23)	TRE (*n* = 45)	Control (*n* = 43)	TRE (*n* = 95)	ADF (*n* = 90)	Control (*n* = 79)	TRE (*n* = 17)	SC (*n* = 15)
Female, %	10 (45.5)	8 (34.8)	21 (47)	18 (42)	66 (69.5)	35 (66.7)	56 (70.9)	NA	NA
Mean age, yrs	41.36 (10.5)	44.17 (4.9)	32.3 (10.5)	31.7 (8.3)	33.56 (6.23)	35.50 (4.42)	34.54 (6.96)	NA	NA
BMI, kg/m2	29.13 (2.64)	30.60 (30.09)	32.2 (3.4)	32.2 (3.2)	26.76 (1.59)	26.12 (2.21)	26.34 (2.73)	29	30
Weight, mean (SD), kg	83.75 (12.71)	89.33 (18.47)	88.9 (10.9)	91.5 (13.6)	74.98 (8.02)	75.32 (8.53)	72.94 (8.00)	81.9	82.3
Waist circumference, cm	104.59 (10.47)	107.09 (9.47)	100.4 (8.2)	102.3 (9.5)	91.54 (4.43)	92.07 (5.29)	92.59 (4.98)	NA	NA
Lean body mass (kg)	57.04 (11.34)	62.10 (11.29)	51.4 (7.9)	52.7 (8.8)	44.54 (4.08)	44.69 (4.57)	43.65 (3.95)	NA	NA
Fat mass (kg)	26.69 (5.35)	27.21 (7.33)	33.8 (7.4)	35.0 (7.2)	30.27 (3.23)	30.58 (3.95)	29.06 (3.64)	NA	NA

### 3.3 The effects of TRE for NAFLD

Cai et al. (57) performed a 4-week study to investigate the effects of TRE and ADF on dyslipidemia, body composition, and body weight in patients with NAFLD. A total of 271 adults were included, with 95 individuals in the TRF group, 90 individuals in the ADF group, and 79 individuals in the control group. The results demonstrated that both TRE and ADF can reduce body weight, fat mass, total cholesterol, and serum triglycerides. There were no significant changes in HDL, fasting insulin, fat-free mass, LDL, liver stiffness, glucose, and systolic or diastolic blood pressure between the TRE and control groups. The authors found that TRE, as well as ADF, were effective diet interventions for patients with NAFLD, with an improvement in dyslipidemia and a reduction in body weight achieved.

Kord-Varkaneh et al. (55) conducted a 12-week study to explore the effects of TRF combined with a low-sugar diet versus a traditional diet for NAFLD. In their study, 52 patients were included, and 3 and 4 patients dropped out of the control group and intervention group, respectively; eventually, 45 patients were included in the analysis. Compared with the traditional diet, TRF combined with a low-sugar diet could significantly reduce the γ-glutamyl transpeptidase, alanine aminotransferase, fibrosis score, aspartate aminotransferase, inflammatory markers of cytokeratin, high-sensitivity C-reactive protein, triacylglycerols, and total cholesterol statistically. The authors concluded that TRF combined with a low-sugar diet can improve lipid, liver, and inflammatory markers, and reduce adiposity. Therefore, TRF combined with a low-sugar diet could be regarded as a promising non-pharmacologic therapy for NAFLD, and long-term adherence should be considered in further studies.

In their 12-month study, Wei et al. (56) compared the effect of TRE with calorie restriction for patients with NAFLD and obesity; 88 patients were included. In total, 81 (92%) and 74 (84%) patients completed the 6-month intervention and entire 12-month intervention, respectively. TRE and CR reduced the intrahepatic triglyceride (IHTG) content by 8.3 and 8.1%, respectively, at the 6-month assessment. TRE and DCR can significantly reduce body weight, metabolic risk factors, and liver stiffness; however, there was no significant difference between the two groups regarding these factors. In addition, regarding the safety of TRE, the authors found that there were no significant differences between the TRE plus CR group and the CR group for adverse events, including appetite change, dyspepsia, constipation, hunger, fatigue, decreased appetite, discomfort in the stomach, and dizziness. Their findings supported that caloric restriction should be added to TRE.

Mack et al. (54) investigated the effects of TRE on patients with NAFLD, comparing it to the standard care (SC) of diet and exercise. The study enrolled 32 NAFLD patients, who were randomly assigned to either the SC or TRE group. The results indicated improvements in both groups in terms of weight, BMI, and total body fat mass reduction. However, the TRE group showed more significant improvements in liver stiffness, liver steatosis, waist circumference, and visceral fat volume compared to the SC group. Additionally, insulin resistance, measured by HOMA-IR, decreased more in the TRE group. Notably, there were no significant differences in dietary energy consumption, activity levels, hunger, or quality of life scores between the two groups. The study concluded that TRE is a viable and more effective approach than standard diet and exercise for treating NAFLD and central adiposity, offering significant improvements in liver health and insulin resistance.

In addition, there were six ongoing studies on TRE for patients with NAFLD that were registered in ClinicalTrials.gov (58). Among them, two trials will explore the effects of TRE on insulin sensitivity, metabolic inflammation, liver steatosis (NCT05220956), and the amount of fat in the liver (NCT04899102). Two trials will investigate the effects of the combination of TRE and a balanced mediterranean diet for glucose metabolism (NCT05866744) and metabolism and inflammation (NCT05968378), and one trial (NCT05332613) will assess the impact of TRE with standard of care lifestyle recommendations (30 min of exercise at least 5 days/week and a mediterranean, hypocaloric diet) on the degree of fat in the liver, measured by magnetic resonance imaging (MRI). One study aims to conduct a prospective study to determine the effects of TRE on 10-year cardiovascular disease risk and the amount of intrahepatic fat in adults with NAFLD via a mobile application (NCT05579158). An overview of the relevant ongoing trials registered at ClinicalTrials.gov is displayed in Table 3.

**Table 3:** Overview of ongoing trials registered at ClinicalTrials.gov.

NCT Number	Study title	Study status	Interventions	Study type	Study design	Start date	Completion date	Location
NCT04899102	Intermittent fasting for NAFLD in adults	Recruiting	Behavioral: time-restricted, intermittent fasting	Interventional	Allocation: NA, intervention model: single_group, masking: none, primary purpose: treatment	2022/2/1	2025/7/31	Massachusetts General Hospital, Boston, Massachusetts, 02114, United States
NCT05220956	Impact of time-restricted feeding in NAFLD	Recruiting	Behavioral: intermittent fasting, behavioral: DGE diet	Interventional	Allocation: randomized, intervention model: parallel, masking: none, primary purpose: screening	2021/10/1	22-Dec	University Medical Center Of The Johannes Gutenberg Univeristy, Mainz, Rheinland-Pfalz, 55,131, Germany
NCT05332613	Diet and meal timing in patients with non-alcoholic fatty liver disease: a pilot study	Recruiting	Behavioral: TRE plus soc., behavioral: soc., behavioral: TRE	Interventional	Allocation: randomized, intervention model: parallel, masking: none, primary purpose: prevention	2022/5/9	2025/12/31	Weill Cornell Medicine, New York, New York, 10,021, United States
NCT05579158	Effects of TRE supported by mobile technology in patients with non-alcoholic fatty liver disease: randomized controlled trial	Recruiting	Behavioral: mobile application and wearable device, behavioral: time-restricted eating	Interventional	Allocation: randomized, intervention model: parallel, masking: none, primary purpose: treatment	2023/2/1	2024/12/31	Hanyang University Seoul Hospital, Seoul, 133,792, Korea
NCT05866744	Effects of time-restricted hypocaloric diet in patients with NAFLD	Recruiting	Other: early time-restricted feeding plus hypocaloric mediterranean diet	Interventional	Allocation: randomized, intervention model: parallel, masking: none, primary purpose: treatment	2023/5/8	2024/7/30	Laiko General Hospital Of Athens, Athens, Attica, 11,527, Greece
			Other: late time-restricted feeding plus hypocaloric mediterranean diet					Agricultural University Of Athens, Athens, Attica, 11,855, Greece
			Other: hypocaloric mediterranean diet without time restriction in feeding					
NCT05968378	Diet, immunometabolism and non-alcoholic fatty liver disease	Recruiting	Behavioral: standard healthy eating advice, behavioral: eTRE plus mediterranean diet	Interventional	Allocation: randomized, intervention model: parallel, masking: none, primary purpose: treatment	2023/6/1	24-Jul	University College Dublin, Dublin, Leinster, D04 V1w8, Ireland

## 4 Discussion

To the best of our knowledge, this is the first systematic review to summarize and analyze all RCTs on the effects of TRE in patients with NAFLD. The findings suggest that TRE may be an effective diet therapy for patients with NAFLD, which can improve liver, lipid, and inflammatory markers.

NAFLD is a heterogeneous disease involving complex interplay between inflammatory mediators, immune cells, and metabolic target tissues of skeletal muscle and adipose (59, 60). It is a huge challenge to develop effective drugs for NAFLD. To date, no effective drugs have gone through late-phase trials and into licensing (4, 6). The main interventions for patients with NAFLD are weight loss and lifestyle intervention (61). Traditionally, diets include a balanced mediterranean diet and/or limiting consumption of saturated fats, fructose, and ultra-processed foods, which focus on the dietary macronutrient composition and have been proven to improve hepatic steatosis and liver biochemistry (62). Recently, accumulating evidence has demonstrated that the timing of energy intake may play a key role in the risk of NAFLD, including night-time eating and irregular meal patterns (63–66). The link between NAFLD and eating habits may be mediated through disruption to circadian rhythms. The benefits of TRE for patients with NAFLD should be investigated.

In our systematic review, Cai et al. (57) found that TRE was well tolerated and related to significantly decreased fat mass, weight loss, and triglycerides. In this study, the parameters of circadian markers, inflammation, steatosis, and liver biochemistry at the baseline and follow-up were not measured, and no change of liver stiffness may be on account of the short intervention period (only 4 weeks). Kord-Varkaneh et al. (55) found that TRF combined with a low-sugar diet can improve lipid, inflammatory, and liver markers, and reduce adiposity for NAFLD. The intervention period was still short (3 months), and a long-term intervention (more than 1 year) may provide a better understanding of the adherence and the chronic effects of the intervention. Wei et al. (56) found that both TRE plus CR and CR were effective for weight loss, with marked reductions in both liver stiffness and IHTG; however, there was no significant difference between the two groups. In this study, the stringent calorie restriction targets might mask the relatively subtle benefits of TRE. Mack et al. (54) demonstrated in their pilot study that TRE is a favorable approach for managing NAFLD and central obesity, showing notably better outcomes in terms of improving liver health, reducing waist size, decreasing visceral fat, and enhancing insulin sensitivity when compared to conventional diet and exercise recommendations. In total, limited evidence demonstrated that TRE may have potential benefits for patients with NAFLD, including weight loss, the reduction of cardiometabolic parameters such as triacylglycerols and total cholesterol, insulin resistance, the reduction of inflammation parameters such as cytokeratin and high-sensitivity C-reactive protein, and an improvement in the live parameters of liver stiffness and IHTG. In addition, in terms of the feasibility and safety of TRE, all four RCTs demonstrated that TRE had good feasibility. Although short intervention can provide relevant results, a longer-term study (e.g., more than 1 year) would be superior for understanding certain factors, including safety and efficacy. However, the intervention time is relatively short (no more than 1 year) in these studies, and a long-term study of TRE should be conducted in further studies. In addition, a cross-sectional study found that TRE may be associated with a lower risk of NAFLD. However, the time of TRE was derived from recall, which may cause bias; the cross-sectional design could not demonstrate causality (67). For the safety of TRE, only one included study reported the AE of TRE, and there were no significant differences between the TRE group and control group for AEs, discomfort in the stomach, appetite change, dyspepsia, constipation, hunger, fatigue, decreased appetite, and dizziness. It seems that TRE is a safe dietary intervention and many previous studies have also demonstrated that TRE is safe. Nevertheless, it is imperative to note that TRE may pose potential side effects, including the risk of orthorexia and sarcopenia. These concerns warrant careful consideration and monitoring during the implementation of TRE interventions (68, 69).

The mechanisms of the benefits of TRE for patients with NAFLD are multiple (16, 19, 70), including: (1) TRE activates pathways in the liver (70, 71). During the long period of daily fasting, the expression and functional states of liver-based sirtuins, ketone bodies (ketogenesis), and adeno-sine monophosphate–activated protein kinase C (AMPK) are increased, which can activate pathways implicated in mediating the benefits of CR, including weight loss, and the improvement in cardiometabolic parameters. (2) TRE improves circadian rhythms (72). TRE can change the temporal pattern activity of the important mediator of the insulin signaling pathway within the liver. (3) The effects of TRE on adipose tissue (73). TRE can increase lipolysis and β-oxidation in adipose tissue. In addition, the overall reduction in free fatty acid in the liver as a result of TRE can reduce inflammation. (4) The effects of TRE on gut microbiota (74). TRE can restore the microbiota-related molecular pathways, including hormonal signaling, neural responses, metabolic regulators, the circadian system, and immune-inflammatory pathways. (5) The effects of TRE on endocrine regulators of metabolism and physiology (75). TRE, particularly early TRE, is related to the most marked improvements in appetite reduction and endocrine regulators, considering the setting of isocaloric intake.

One included study (55) investigated the combination of eating window (TRE) and dietary macronutrient composition (a low-sugar diet) for patients with NAFLD. Whether this combination is more effective should be explored in further studies, including but not limited to the combination of TRE and a low-sugar diet, TRE and a balanced mediterranean diet, and TRE and a low and very low carbohydrate diet. In addition, previous studies have demonstrated that exercise-based interventions have benefits for patients with NAFLD and suggested their use in the management of NAFLD. However, there is no study investigating the effects of TRE combined with exercise for patients with NAFLD. The combined interventions should be explored in further studies.

This systematic review presents several strengths. It is the inaugural comprehensive analysis that collates and evaluates all clinical studies on the effects of TRE in patients with NAFLD. Our findings indicate the potential health benefits of TRE for NAFLD patients, including weight reduction, decreased cardiometabolic parameters such as triacylglycerols and total cholesterol levels, and improvements in liver health indicators, notably liver stiffness and IHTG levels. There are some limitations in this systematic review. First, although meta-analysis was initially planned, the types of interventions (TRE with and without CR, TRE plus a low-sugar diet), the interventions time (from 4 weeks to 1 year), the primary measured outcomes, and statistical heterogeneity make meta-analysis inappropriate. Second, there are only four RCTs on the effects of TRE in NAFLD, and none of the RCTs compared “TRE without additional dietary intervention” to control conditions. Therefore, the superiority of TRE itself cannot be deduced. Notably, several ongoing trials, including those investigating the synergistic effects of TRE with a balanced mediterranean diet, promise to yield novel insights into this domain, potentially highlighting augmented benefits for NAFLD patients.

In conclusion, TRE might be an effective dietary intervention for improving liver parameters and reducing the cardiometabolic risk in patients with NAFLD. However, it is premature to recommend TRE for patients with NAFLD, given the current limitations in evidence. Thus, further high-quality research is imperative to broaden our understanding of TRE’s benefits in the context of NAFLD. The results from ongoing clinical trials are anticipated to offer additional insights into the efficacy of TRE in managing NAFLD.

## Data availability statement

The original contributions presented in the study are included in the article/supplementary material, further inquiries can be directed to the corresponding authors.

## Author contributions

XL: Data curation, Formal analysis, Methodology, Project administration, Supervision, Validation, Writing – original draft, Writing – review & editing. SW: Conceptualization, Data curation, Investigation, Methodology, Writing – original draft, Writing – review & editing. JH: Formal analysis, Funding acquisition, Project administration, Resources, Validation, Visualization, Writing – original draft, Writing – review & editing.

## Footnotes

^1^https://clinicaltrials.gov/
